# High-Resolution Contrast-Enhanced Ultrasound with SRCEUS for Assessing the Intrahepatic Microvasculature and Shunts in Patients with Hereditary Haemorrhagic Teleangiectasia (Osler’s Disease)

**DOI:** 10.3390/life15101631

**Published:** 2025-10-20

**Authors:** Irmgard Maria Sieber, Friedrich Jung, Ernst Michael Jung

**Affiliations:** 1 Interdisciplinary Ultrasound Department and Institute for Radiology, University Hospital Regensburg, 93053 Regensburg, Germany; 2 Institute of Biotechnology, Molecular Cell Biology, Brandenburg University of Technology, 01968 Senftenberg, Germany; dihkf@saarmail.de

**Keywords:** Osler’s disease, shunts, superresolution CEUS (SR-CEUS), multiparametric tissue imaging (M-Ref)

## Abstract

The aim of this retrospective clinical pilot study is to evaluate multiparametric ultrasound liver parenchyma assessments in the diagnosis of Osler’s disease, and to detect micro-shunts using SRCEUS with quantifications at the capillary level. **Material/Method:** All examinations were performed by an experienced examiner with a multi-frequency probe on a high-resolution matrix ultrasound device (SC 7-1U), convex probe (Mindray A 20), and were stored digitally in the PACS system. Vascular ultrasound was performed using colour-coded Doppler ultrasound (CCDS) and ultrasound microangiography (UMA). The recent M-Ref tool was utilised for the purpose of liver tissue characterisation, encompassing the domains of shear wave elastography, fat evaluation, and viscosity. Dynamic CEUS, HiFR CEUS, and SR CEUS were performed after the intravenous bolus injection of 1–2.4 mL of ultrasound contrast agent (SonoVue^®^). Measurements of SR CEUS capillary changes were performed independently by PACS-stored digital cine loops up to 5 s. **Results:** In the context of angiomas or haemangiomas, the initial contrast enhancement of echogenic or almost echogenic foci within 25 s without late wash-out was observed in 5/10 cases. In the evaluation of microvasculature, the presence of capsule-proximal shunts in Osler’s disease was observed, resulting in the identification of increased numbers of dilated capillaries within both peripheral and central shunts. In the control group, general liver tissue changes (20 cases) were observed in instances of inflammation (3/20 cases), peripherally in 4/20 cases with micro-shunts in altered parenchyma. In the context of multiparametric ultrasound, 16 out of 30 cases exhibited elevated fibrosis values, with a maximum recorded as high as 1.7 m/s, and in 13 out of 30 cases, there was an increase in fat values up to 0.65 dB/cm/MHz, indicative of moderate steatosis. Additionally, in seven cases, there was an increase in viscosity values up to 2.7 Pa·s, suggesting reactive changes. **Conclusions:** Recent advancements in medical imaging technology, specifically SR CEUS contrast ultrasound imaging, have led to the development of novel diagnostic tools that facilitate the evaluation of tissue and haemodynamic changes, in addition to capillary alterations, associated with Osler’s disease.

## 1. Introduction

Contrast-enhanced ultrasound sonography (CEUS) has become increasingly important in recent years [1,2,3,4,5,6,7,8,9,10,11,12,13,14,15,16,17,18]. It allows dynamic intrahepatic microvascular changes to be visualised up to the capillary level. The major advantage of dynamic CEUS is that changes in blood flow and its dynamics in the liver can be assessed. It provides detailed information from the early arterial phase, after just 9–15 s, to a late phase of 6–7 min [4,19,20,21,22,23,24,25].

**Figure 1:**
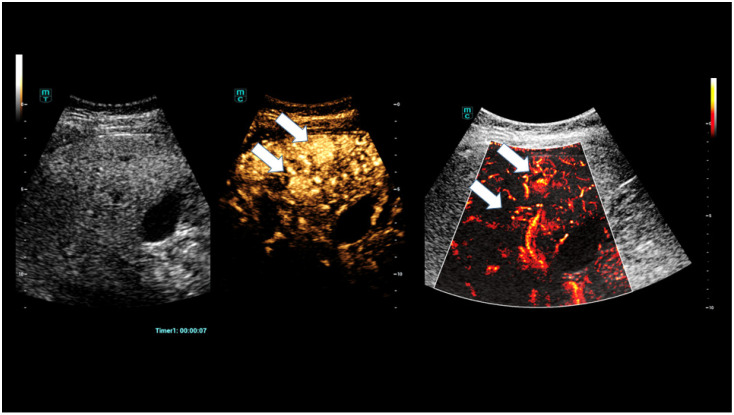
CEUS and SR CEUS after injection of 1.4 mL ultrasound contrast agent with irregular hyperenhancement of the liver and early nodular enhancement of high-flow haemangiomas (arrows) and micro-shunts.

A particular advantage of modern CEUS using sulphur hexafluoride microbubbles (SonoVue^®^/Bracco) is that there is only a low risk to the kidneys, thyroid, and other organs when the contrast agent is administered by venous bolus injections. There is a very low risk of an allergic reaction, less than 1:10,000. Modern high-performance ultrasound devices enable high amplification in the special contrast agent technique at low power and a significantly reduced mechanical index (MI less than 0.2) through oscillating microbubbles (Figure 1) [4,17].

This allows for detailed dynamic analyses at the capillary level. This has led to CEUS of the liver being used increasingly worldwide. It is not only to detect parenchymal changes, but also to clarify complicated cystic changes, solid lesions, and benign and malignant tumours, considering the EFSUMB liver and non-liver guidelines [4,17,21,22].

Multicentre clinical liver studies have firmly established the high diagnostic value of CEUS of the liver. International guidelines for the use of CEUS of the liver include the results of these multicenter studies and give the first clinical recommendations [4,6,7,13,14,15,16,18,26].

**Figure 2:**
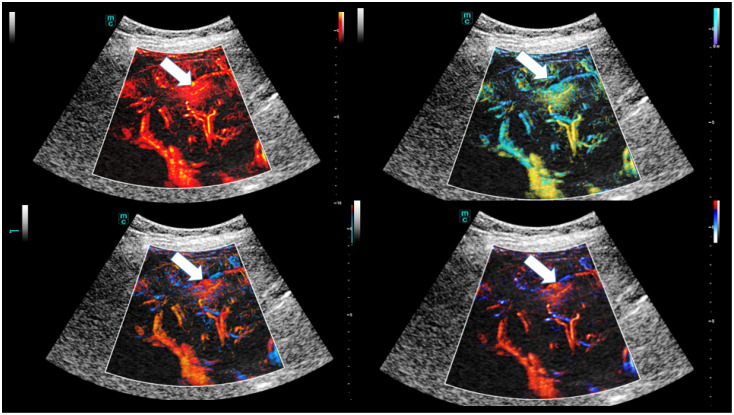
Different modalities of SR CEUS after injection of 1.4 mL ultrasound contrast agent with irregular hyperenhancement of the liver and early nodular enhancement of high-flow haemangiomas (arrows) and micro-shunts. Visualisation by dynamic SRCEUS (red colours), velocity (yellow and green), directional (blue and red), and bi-directional (blue, red, orange).

The detailed assessment of dynamics is also being expanded through accurate perfusion and dynamic evaluation of SR CEUS with four different tools, including dynamic, velocity, directional, and bi-directional (Figure 2).

**Figure 3:**
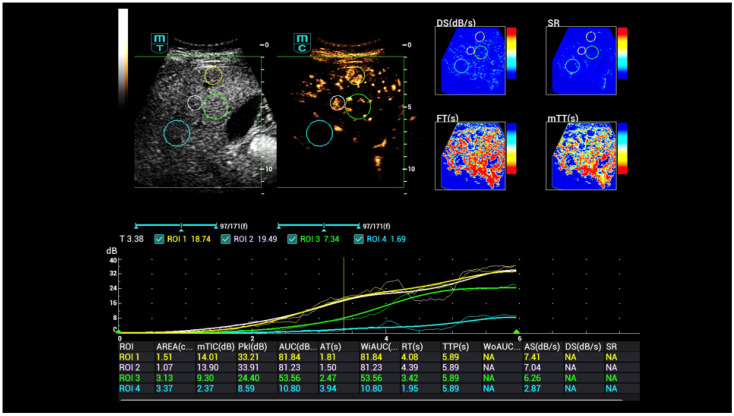
CEUS perfusion evaluation after injection of 1.4 mL ultrasound contrast agent with irregular hyperenhancement of the liver and early nodular enhancement of high-flow haemangiomas and micro-shunts. In the early arterial phase, there is strong contrast enhancement into the areas of micro-shunts.

Initially, time–intensity curve analysis (TIC) was used, which was then expanded to include false-colour analyses and parametric contrast-enhanced sonography (parametric CEUS, Figure 3). TIC analysis could be performed by digital stored cine loops with different haemodynamic parameters like time-to-peak (TTP), peak intensity (PI), wash-in rate, and area-under-the-curve (AUC) [1,11,16].

Furthermore, more detailed perfusion analyses with a variety of perfusion parameters are now available, which were previously only known from other imaging techniques in relation to increased or decreased perfusion, particularly in the neurocranium. Preliminary investigations have already shown that intrahepatic shunts in Osler’s disease pose a risk in terms of spontaneous bleeding [3,27,28,29,30,31].

**Figure 4:**
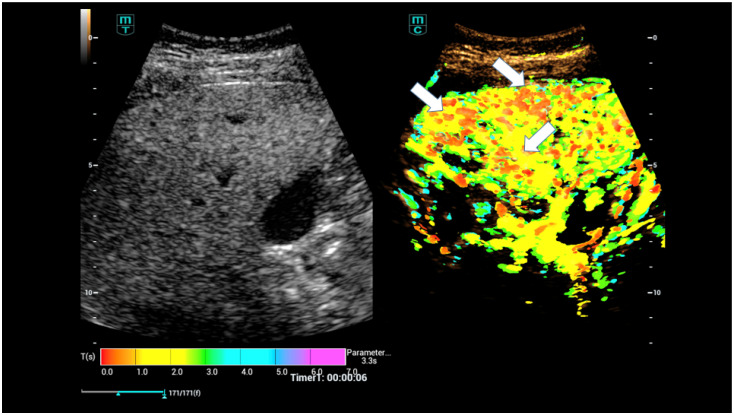
CEUS parametric evaluation after injection of 1.4 mL ultrasound contrast agent with irregular hyperenhancement of the liver and early nodular enhancement of high-flow haemangiomas (arrows) and micro-shunts with higher levels for time-to-peak (TTP) and area-under-the-curve (AUC).

Incipient shunts are usually located close to the capsule (Figure 4). As Osler’s disease progresses, the central liver vessels and the hepatic artery dilate due to the increase in blood flow. In advanced stages, flow changes also occur in the portal vein (V portae), ultimately leading to a high proportion of flow in the hepatic veins. Changes in the liver must be detected at an early stage at the capillary level. High-resolution spiral computed tomography angiography (CTA) and special 3D examinations in magnetic resonance imaging (MRI), known as special MRI angiography techniques (MRA) with reference to so-called micro-shunts, have made this possible [3,27,32,33].

In the further course, angiomatous tumours, haemangiomas, high-flow angiomas and, in rare cases, angiosarcomas may also develop. Contrast agent ultrasound technology is advancing rapidly. High frame rate technology (HiFR) and super-resolution (SR CEUS) technologies are being developed in line with superselective angiography techniques in order to be able to produce a dynamic, detailed image of the vessels [2,4,26].

**Figure 5:**
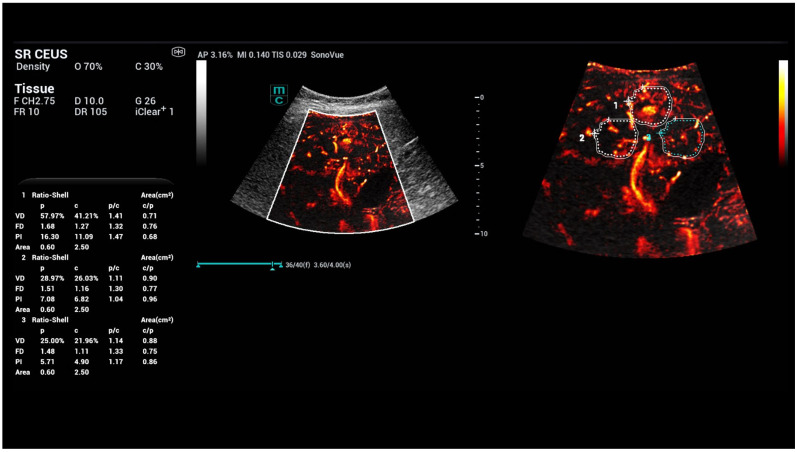
SRCEUS perfusion evaluation after injection of 1.4 mL ultrasound contrast agent with irregular hyperenhancement of the liver and early nodular enhancement of high-flow haemangiomas and micro-shunts. In the early arterial phase, there is strong contrast enhancement in the areas of micro-shunts with capillary changes in the microvascular density and ratio shell (area 1).

Digital subtraction angiography emphasises the vascular arterial component and can therefore depict shunts in detail in a dynamic manner. However, it is associated with high radiation exposure. The selective imaging of micro-shunts requires an examiner with extensive experience. This raised the question of the extent to which peripheral venous injection of only small doses of ultrasound contrast medium (between 1 mL and a maximum of 2.4 mL) can be used to detect early changes in Osler’s disease and whether the new super-resolution (SRCEUS) technique can be used to reliably detect micro-shunts (Figure 5).

It is also vital to evaluate the extent to which capillary changes can be quantified in the future. In addition, these changes should be linked to a multiparametric liver parenchyma assessment for the first time [2,26].

This pilot study aims to demonstrate the possibilities of multiparametric ultrasound liver parenchyma assessments in Osler’s disease and the detection of micro-shunts using SRCEUS with quantifications at the capillary level.

## 2. Materials and Methods

This retrospective study includes outpatients from the University of Osler’s centre for liver ultrasound examination under risk management for liver tissue bleeding from possible intrahepatic shunts or angiomas after successful sclerotherapy for nose bleeding due to Osler’s disease. The evaluation encompassed a DICOM dataset of a total of *n* = 30 patients aged between 25 and 82 years with a mean age of 52 years ± 14.9 years. The reference group included 20 patients with follow-up of known liver tissue changes, determined by ultrasound 6 and 12 months prior, and 10 patients with known liver tissue changes and Osler’s disease, determined by ultrasound in follow-ups after one year. The follow-ups were performed using SR CEUS, following the same protocol as in the preliminary studies [26]. Written informed consent was obtained from patients for all examinations using CEUS. The Ethics Committee of the University Hospital Regensburg has approved contrast-enhanced sonography and multiparametric ultrasound for the diagnosis of Osler’s disease (24-3773-104).

All examinations were performed by an experienced examiner with more than 3000 examinations per year and over 20 years of experience. The results were stored digitally in the PACS system. All the examinations were performed with a multi-frequency probe on a high-performance ultrasound device (SC 7-1U convex probe, Mindray A 20, Mindray, China, Shenzhen). Appropriate protocols were documented in B-mode liver sonography using various standard techniques. In particular, the volume status of the inferior vena cava and the representation of the hepatic veins next to the hepatic hilum with the portal vein and hepatic artery were recorded.

If present, local parenchymal changes, cystic lesions, or solid components were documented in B-mode. The haemodynamic examination of the hepatic vessels was then documented. This included recording the hepatic vein flow and angle-corrected flow measurement of the portal vein (V portae) and the hepatic artery.

In accordance with Osler’s disease recommendations, the diameter of the hepatic vessels at the hilum was also determined. If the portal vein showed clearly turbulent flow and the hepatic artery showed elongation and dilatation, a search for possible shunts was performed using colour-coded Doppler ultrasound (CCDS). This resulted in a targeted examination at the transition point to the hepatic veins. The liver parenchyma was assessed in the fundamental B-mode in comparison with the adjacent kidney tissue. An increase in the ultrasound intensity of the liver compared to normal kidney parenchyma was interpreted as proportional steatosis.

Multiparametric imaging also enabled the determination of liver compaction using shear wave elastography for liver stiffness evaluation. In addition, liver steatosis could be determined using the attenuation function (USAT) [1,2].

**Figure 6:**
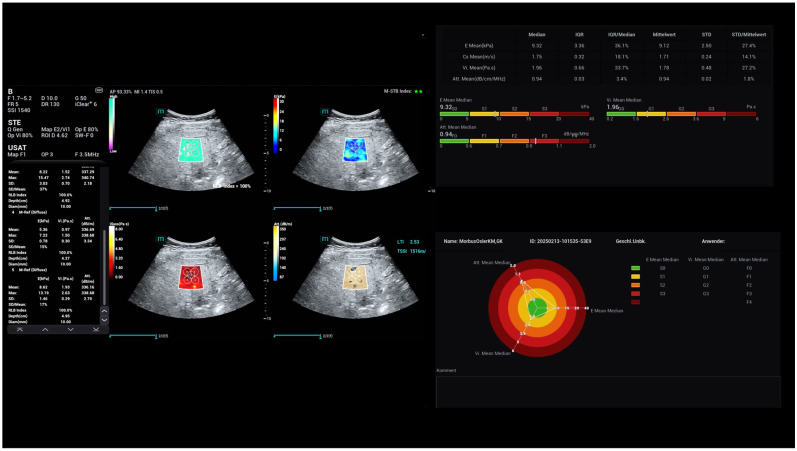
Characterisation of the liver tissue of a young female patient with tissue changes, considering a fatty liver (0.9 dB/cm/MHz) and moderate fibrosis (1.7 m/s).

. Another new module used for the first time is viscosity determination. This new module from M-Ref was used to specifically assess inflammatory tissue changes, if present, or the liver parenchyma (Figure 6).

**Figure 7:**
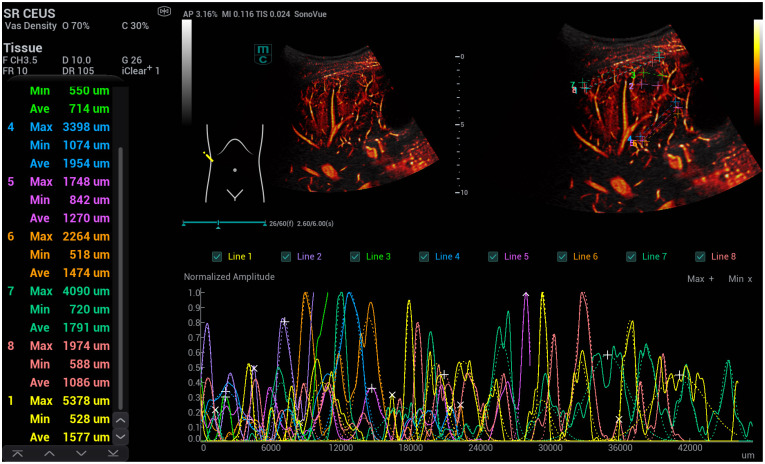
SRCEUS perfusion evaluation after injection of 1.4 mL ultrasound contrast agent with irregular hyperenhancement of the liver and early nodular enhancement of high-flow haemangiomas and micro-shunts. In the early arterial phase, there is strong contrast enhancement in the areas of micro-shunts with capillary changes in the vascular diameters (lines 1, 2, 5).

**Figure 8:**
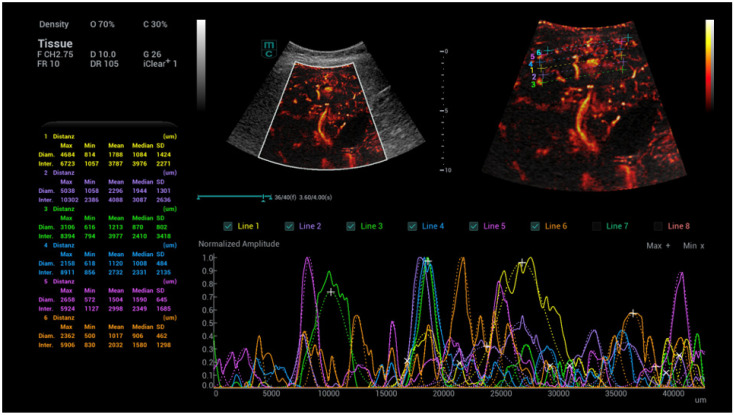
SRCEUS perfusion evaluation in another case after injection of 1.4 mL ultrasound contrast agent with irregular hyperenhancement of the liver and early hyperenhancement of capsular micro-shunts. In the early arterial phase, there is strong contrast enhancement in the areas of micro-shunts with capillary changes of the vascular diameters (line 1) near the capsular structures in comparison to the central vascular structures (line 5).

**Figure 9:**
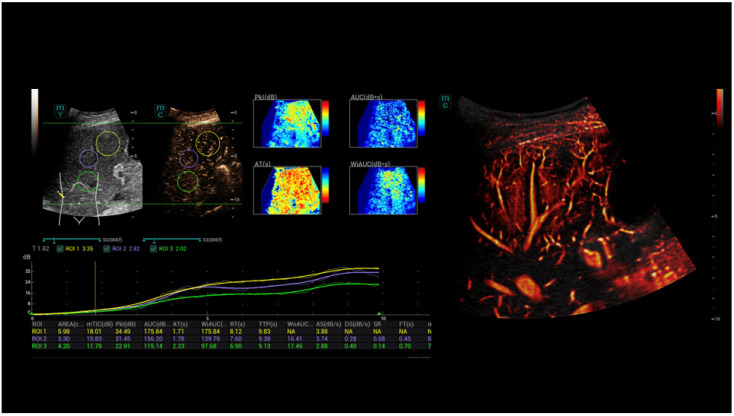
SR CEUS and CEUS perfusion and after injection of 1.4 mL ultrasound contrast agent with irregular hyperenhancement of the liver and early nodular enhancement of high-flow haemangiomas and micro-shunts. Visualisation of the early time–intensity curve (TIC) hyperenhancement of the shunts by dynamic SRCEUS (red and orange), as well as by colour maps of peak enhancement (PKI) and wash-in area-under-the-curve (WiAUC) (red and yellow).

Ultrasound microangiography (UMA) was used for a detailed assessment of intrahepatic shunts. The higher frame rate and reduced angle dependence of the UMA allowed specific areas with increased microvascularisation to be examined. Three different ultrasound microangiography (WVA) techniques were used, which included colour-coded Doppler ultrasound (CCDS), power Doppler, and a super-resolution CEUS examination (Figure 7, Figure 8 and Figure 9).

**Figure 10:**
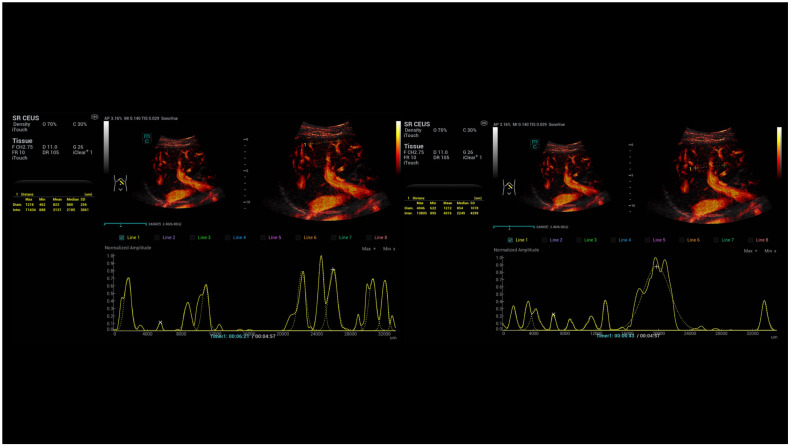
Diameter measurements of capillary vascalary structures near the liver capsular structure and in the center of the liver using a machine integradet SR CEUS quantification software. Independent evaluation by the stored dynamic SR CEUS cine loops over 5 s with manually adapted area’s near the capsular structures in comparison to the centre with peripheral capillary dilation in a case of Osler’s disease. CEUS in arterial phase after bolus injection of 2.0 mL ultrasound contrast agent (SonoVue) using a convex multifrequency probe (1–7 MHz).

**Figure 11:**
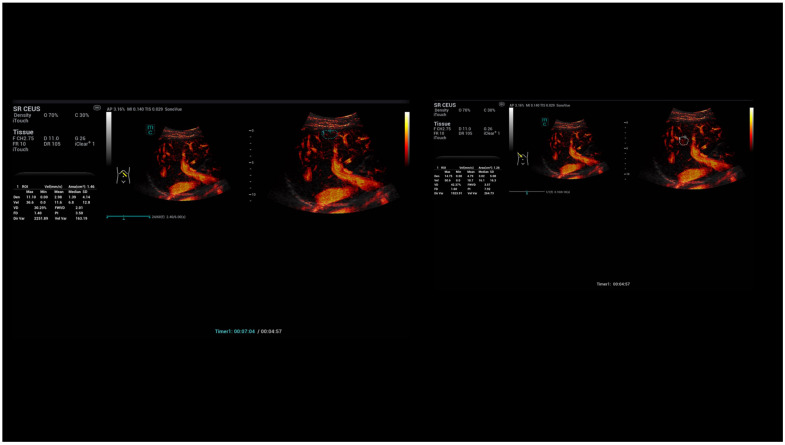
Area measurements of capillary vascalary structures near the liver capsular structure and in the center of the liver using a integradet SR CEUS quantification software. Independent evaluation by the stored dynamic SR CEUS cine loops over 5 s with manually adapted area’s near the capsular structures in comparison to the centre with peripheral capillary dilation in a case of Osler’s disease. CEUS in arterial phase after bolus injection of 2.0 mL ultrasound contrast agent (SonoVue) using a convex multifrequency probe (1–7 MHz).

For the measurement of the capillary vascular structures, linear measurements and additional area measurements were carried out. Both methods were then used to measure close to the capsule and in the centre of the liver (Figure 10 and Figure 11).

Prior to each CEUS examination, patients were provided with written information. The risks of contrast agent administration were discussed. The risk of an allergic reaction was classified as less than 1:10,000 according to the guidelines. For the bolus contrast agent application, intravenous access was established in the cubital vein. A weight-adjusted bolus (1 mL to a maximum of 2.4 mL of ultrasound contrast agent with 10 mL of saline solution (NaCl)) was administered. The ultrasound contrast agent SonoVue is available in Europe. This involves the administration of sulphur hexafluoride microbubbles. At a low mechanical index of less than 0.2 (MI < 0.2), oscillating microbubbles in the sound region cause dissolution down to the capillary level. Compared to conventional ultrasound, the resolution is 30 times higher in detail. Digital raw data was stored for the contrast agent examinations. This must be available in DICOM format and can then be post-processed.

Data are reported as arithmetic means with standard deviations for continuous variables and were analysed by a two-sided Student’s *t*-test for unpaired samples. A *p*-value of less than 0.05 was considered significant.

## 3. Results

The observed changes included intrahepatic shunts—visible by CCDS or UMA- and peripheral shunts near the capsular structures, micro-shunts, which were only detectable by dynamic CEUS and SR CEUS, and early capsular hyperaemia, particularly in liver segments VII, VIII, and IVa.

The criteria for macro-shunts were modified in 4/10 cases, with alterations made to the following parameters:

- The flow of the hepatic artery was characterised by dilatation > 5 mm.
- The resistance index (RI) was reduced, with a value of <0.55.
- The flow of the portal vein increased proportionally, with a velocity > 30 cm/s.
- The flow of the hepatic veins increased proportionally or was continuous.

In the context of angiomas with early regular arterial hyperenhancement or haemangiomas with nodular rim enhancement without wash out, the initial contrast enhancement of echogenic or almost echogenic foci within 25 s without late wash-out was observed in 5/10 cases. In evaluating capillary diameters, the presence of capsule-proximal shunts in Osler’s disease was observed, resulting in the identification of increased numbers of dilated vessels within both peripheral and central shunts. In the control group, comprising 20 cases, general hyperemia was only evident in instances of inflammation (3/20 cases), peripherally in 4/20 cases with micro-shunts in altered parenchyma. In the context of multiparametric ultrasound, 16 out of 30 cases exhibited elevated fibrosis values, with a maximum recorded as high as 1.7 m/s. In 13 out of 30 cases, there was an increase in fat values up to 0.65 dB/cm/MHz, indicative of moderate steatosis. In seven cases, viscosity values increased up to 2.7 Pa·s, indicating reactive changes.

In the comparative measurements by linear observation directly near the capsule, the detection of peripheral, capsular near micro-shunts in Osler’s disease revealed additional and multiple vessels in the terminal stroma. The same could also be detected in adapted areas. Central shunt formation also increased the number and density of vessels to be detected with SRCEUS. The results were highly significant compared to the control group. Table 1 clearly shows the means with standard deviations for diameters and area measurements of the capillary vascular structures near the liver capsule and centrally in the liver for patients with and without Osler’s disease.

The modified examination protocol was designed to ensure that, after bolus administration of the ultrasound contrast agent, the contrast agent accumulation was first documented using conventional ultrasound. If, within the early arterial phase after 9 s to 25 s, there was increased contrast agent accumulation in an area of the liver, it was a clear indication of possible shunt formation. In such cases, the conventional contrast agent technique was immediately switched to HFR CEUS and then to super-resolution CEUS (SRCEUS).

In order to be able to conclusively classify possible solid lesions, documentation was carried out in individual sequences up to a late phase of 6 min. During this latter contrast agent phase, sequences with Super-Resolution CEUS (SRCEUS) were also documented intermittently. This was to conclusively assess possible micro-shunts.

Two experienced examiners independently read the stored images using integrated software and reached a consensus. In addition, parametric false colours and a time–intensity curve with a maximum evaluation time of 10 s were used in the early arterial phase. Compared to the surrounding liver structure, early high contrast in the arterial phase was shown in red and orange.

A slightly increased perfusion region was coded with colours ranging from yellow to green compared to normal liver perfusion, and the liver tissue was displayed in blue to violet in the background.

Finally, based on the stored images in the areas of possible shunts, changes in capillary diameter were additionally measured using predefined lines or individually adapted regions of interest (ROIs). With uniform capillaries, the curve was low-modulated. In the case of strongly fluctuating vessel diameters under increased circulation, curves with increased modulation and high amplitude were observed.

## 4. Discussion

This pilot study on high-resolution CEUS in patients with Osler’s disease builds upon our own findings from preliminary investigations [3,26,27,28,29,30,31,32,33]. Compared to high-resolution superselective catheter angiography, CEUS can be used to detect micro-shunts. The advantage of using new ultrasound technologies, HiFR and SRCEUS, is that they can capture dynamics down to the capillary level.

In SRCEUS, the background can be subtracted, and a measurement function can be employed to evaluate capillary changes in terms of diameter alterations, enabling micro-shunts to be analysed in detail for the first time. Changes in macro blood flow typically accompany changes in the micro blood flow in Osler’s disease [3,27,28,29,30,31,32,33].

Early detection of micro-shunts on the liver capsule can avoid unnecessary risks to patients and enable a better assessment of the risk of bleeding. Therefore, targeted liver biopsies should be avoided in the capsular region. As the disease progresses, these micro-shunts can then lead to the formation of smaller fistulas. In the advanced stages of Osler’s disease, the hepatic artery can be seen to dilate over time. Further progression of the disease also leads to an earlier and higher flow rate in the portal vein, which ultimately results in a higher flow in the hepatic veins. Partial angiomatous changes may also occur as the disease progresses. This can lead to the formation of so-called high-flow haemangiomas. The development of malignant angiomatous changes, such as angiosarcoma, is much rarer.

In the stepwise multiparametric diagnosis of patients with Osler’s disease, the examination begins with a fundamental B-mode ultrasound. The focus here is on evaluating the surface and shape of the liver, as well as an initial assessment of the tissue structure and vascular architecture. The next step in multiparametric ultrasound imaging is a haemodynamic assessment of the hepatic veins in relation to triphasic, biphasic, or monophasic flow. The filling status of the inferior vena cava must also be determined. In addition, the extent of increased or decreased heart pulsation must be evaluated.

Examination of the portal vein may also indicate enlargement of the portal vein. Dilatation is an indirect indication of potential fistula formation in the sense of a macro-shunt. The assessment of the hepatic artery in B-mode is primarily involved in evaluating elongation or kinking. Colour-coded Doppler sonography can be used to perform a haemodynamic assessment in the different flow areas of the venous system, the hepatic artery, and the portal vein.

According to preliminary examinations and the results of other working groups, the typical changes in elongation and dilatation of the hepatic artery are usually greater than 4 mm. There is also an increase in flow velocity, particularly an increased end-diastolic velocity, and a reduction in the resistance index (RI). The hepatic veins undergo changes in terms of the proportions of possible shunt flow, shifting increasingly from a triphasic flow to a biphasic flow and finally to a monophasic flow.

An increasingly dilated portal vein is a sign of increasing shunt formation with more blood flow volume. This can lead to audible flow components up to a continuous flow. Micro-shunts cannot be detected linearly using colour-coded duplex sonography alone, but can now be detected using dynamic HiFR CEUS and SRCEUS for the first time [2,26].

Marco-shunts can lead to the formation of fistulas and intrahepatic aneurysms or angiomatous tumours, and can also result in pronounced perihepatic malformations. CEUS can detect even the smallest micro-shunts at an early stage. Furthermore, it can use wash-in and wash-out kinetics to distinguish the extent to which there is early contrast enhancement of the portal venous and venous liver vessels and whether fistula formation is indicated. Small angiomatous convolutions can also be detected at an early stage. Aneurysms become easier to detect.

Furthermore, the HiFR and SRCEUS techniques enable a detailed capillary assessment of shunts with transition to haemodynamic changes of macro-shunts. Initial quantifications indicate that changes in capillary density and capillary diameter occur within shunts.

The results of the multiparametric assessment of the liver parenchyma using elastography and viscosity measurement showed that in individual cases with pronounced shunt formation, there may be an increase in shear wave elastography, and that circumscribed areas with angiomatous tumours show higher viscosity and irregular vascular pattern. In particular, it remains to be established to what extent the new high-resolution ultrasound contrast agent techniques of HiFR and SRCEUS can contribute to the more targeted implementation of interventional procedures [2,16,25,26,34].

The following limitations should also be noted: first, this method requires a highly experienced expert examiner, and second, it necessitates high-end technology for implementation. The small number of cases of Osler’s disease with intrahepatic changes does not allow for definitive results. Additionally, with other high-end systems, the dynamic evaluation of intrahepatic shunts is possible from the early arterial phase after 10 s to the late phase of CEUS up to 6 min. For contrast-enhanced CT and MRI, contrast documentation is only possible for some time points, such as in the arterial or portal venous phase. The radiation exposure for a perfusion CT would be overly high for the examination of younger patients. The risks associated with contrast agents used in CT and MRI could be an appropriate topic for conducting a multicentric evaluation of bleeding risks from intrahepatic shunts in Osler’s disease using CEUS and measuring capillary changes with SRCEUS [12,13,14,15,26].

Recent progressions in the domain of medical imaging technology, particularly in the field of contrast ultrasound imaging (SR CEUS) and multiparametric liver tissue characterisation (M-Ref) [35], have precipitated the formulation of innovative diagnostic tools, which in turn enable the appraisal of tissue and haemodynamic changes, in conjunction with capillary alterations, associated with Osler’s disease. If changes in the local viscosity are to be a diagnostic tool to differentiate between benign and malignant angiomas, they must be evaluated by multicentric studies. Actually, the own research group could publish the first results of evaluations of the dynamic tumour microvascularization by SRCEUS [26] and liver tissue changes using M-Ref [2,35]. These results were now integrated into international clinical evaluation studies and could be used for multicentric evaluations of Osler’s disease in the future.

The study’s primary conclusion was that SR CEUS facilitates quantitative detection of shunts and substantial disparities in capillary dimensions among patients with hereditary haemorrhagic telangiectasia (Osler’s disease, HHT) and those without HHT who are suffering from liver diseases.

## Figures and Tables

**Table 1:** Comparison of Osler’s disease (*n* = 10) and the control group (*n* = 20). For the diameter (μm) and area (cm^2^) measurements of the capillary vascular structures near the liver capsule and centrally in the liver, three measurements were taken per patient (*n* = 30 Osler group, *n* = 60 for the control group).

	Osler’s Disease	No Osler’s Disease	*p*-Value
N = patient	*n* = 10	*n*= 20	
N = number of measurements	*n* = 30	*n* = 60	
diameter: close to the capsule	5133.7 ± 1456.6	1972.3 + 399.2	6.18989 × 10^−13^
diameter: at the level of the portal vein	2175.3 ± 417.5	2890.7 + 606.9	3.00779 × 10^−11^
area: close to the capsule	68.9 ± 8.8	8.9 + 1.99	1.4051 × 10^−26^
area: at the level of the portal vein	35.8 ± 7.73	16.8 + 3.66	8.29298 × 10^−15^

## Data Availability

All original data are stored at the server of the Institute for Radiology of the University of Regensburg.
